# Glasgow Prognostic Score and Outcomes in Elderly Head and Neck Cancer Surgery With Free-Flap Reconstruction: A Retrospective Study

**DOI:** 10.7759/cureus.74699

**Published:** 2024-11-28

**Authors:** Keitaro Nagano, Kiyomi Kuba, Masami Osaki, Akio Hatanaka, Mutsuko Hara, Kazue Manaka, Shingo Kinoshita, Mitsumura Kazuhiro, Ryohei Mukae, Masaya Umino

**Affiliations:** 1 Department of Otolaryngology, Head and Neck Surgery, Ageo Central General Hospital, Ageo, JPN

**Keywords:** elderly patients, free flap-oral cancer, geriatric assessment, glasgow prognostic score, glim criteria, gps, head and neck cancer, postoperative complications, prognosis, reconstructive surgery

## Abstract

Background

Determining good candidates for head and neck cancer surgery in elderly patients requires consideration of various factors, such as overall health and social background, yet specific evaluation guidelines are lacking. The Glasgow Prognostic Score (GPS) is a marker used to assess nutritional status and prognosis in cancer patients.

Objective

This study aims to evaluate the association between the GPS and both the prognosis and postoperative complications in reconstructive surgery cases for head and neck cancer in patients aged 80 and over.

Materials and methods

A total of 22 patients aged 80 and over who underwent reconstructive surgery in our department between January 2011 and December 2023 were included. Patients were divided into three groups based on their preoperative GPS scores (0, 1, and 2 points). The overall survival was evaluated using the Kaplan-Meier method. The Cox proportional hazards model was used to analyze the association between GPS and prognosis, adjusting for confounders such as malnutrition, primary site, and comorbidities. The association between GPS and perioperative complications classified as Clavien-Dindo grade 3 or higher was also examined.

Results

The mean age was 82.2 years, and 20 (90%) of the subjects had comorbidities. Higher GPS scores were associated with lower survival rates, and GPS was an independent prognostic factor. There was no significant association between GPS and perioperative complications of grade 3 or higher.

Conclusion

The GPS is a useful prognostic indicator in elderly patients undergoing reconstructive surgery for head and neck cancer. However, a comprehensive evaluation such as the Geriatric 8, along with more inclusive malnutrition criteria, is recommended for an overall assessment of good candidates for the procedure.

## Introduction

As a consequence of global aging, the demand for cancer surgery is expected to increase. According to recent reports by the International Agency for Research on Cancer (IARC) and the American Cancer Society (ACS), there were approximately 20 million new cancer cases in 2022, projected to reach 35 million by 2050, a 77% increase from 2022. This rise is attributed to population growth and aging [1].

Decisions regarding highly invasive surgery for the elderly should not be based solely on age but should carefully consider various factors, such as effectiveness, safety, postoperative quality of life, and support system [2]. When considering surgery for the elderly, the importance of functional evaluations such as Comprehensive Geriatric Assessment (CGA) has been reported [3]. However, CGA and other functional evaluation methods come with a number of challenges [4,5]: (1) complexity and time required for evaluation; (2) variation in implementation methods depending on facilities and medical personnel, making standardization difficult; (3) difficulty in obtaining cooperation due to patient fatigue and cognitive decline during evaluation; (4) necessity of multidisciplinary cooperation, making implementation difficult in facilities with limited resources; (5) screening tools such as Geriatric 8 (G8) [6] are rapid and simple but not applicable to all elderly patients; and (6) interpreting results and determining surgical benefits still largely depend on the experience and judgment of medical personnel. Therefore, the guidelines for pre-treatment evaluation and recommended treatment intensity in the elderly are currently lacking.

Moreover, studies that hypothesize and verify specific factors in cases of free tissue transfer reconstructive surgery (hereafter referred to as reconstructive surgery) in elderly patients are almost nonexistent. Therefore, we focused on the Glasgow Prognostic Score (GPS), which is feasible for retrospective review of medical records and practical implementation. The GPS is one of the nutritional indicators first reported in 2003 [7]. This study aims to investigate the relationship between systemic inflammatory response and prognosis in cancer patients, particularly demonstrating that the GPS is associated with prognosis in terminal cancer. In fact, subsequent studies have reported that the GPS is useful for predicting survival in cancer patients, and it has been shown to be a reliable indicator across various tumor types and treatment conditions [8-11]. The test is simple, with scores of 0, 1, and 2 based on blood levels of C-reactive protein and albumin, with higher scores indicating poorer prognosis.

In this study, we examined the usefulness of the GPS in determining the suitability of reconstructive surgery for elderly patients. The purpose of this study is to evaluate the validity of using the GPS to individually assess prognosis and predict complications in order to assist with decision-making in regard to highly invasive treatment in elderly patients.

## Materials and methods

This study employed a retrospective cohort design to evaluate the impact of the Glasgow Prognostic Score (GPS) on elderly patients undergoing free-flap reconstructive surgery. The design was selected for its ability to track long-term outcomes and assess GPS as a prognostic factor in this patient population.

Participants were 22 patients aged 80 and over who underwent free-flap reconstructive surgery at the Department of Otolaryngology, Ageo Central General Hospital, between January 2011 and December 2023. Medical records were reviewable from 2011 onward through the electronic medical record system. No exclusion criteria were applied, allowing for a comprehensive assessment of patient conditions and outcomes related to the GPS.

Data were collected retrospectively by reviewing electronic health records, including patient demographics, Eastern Cooperative Oncology Group performance status (ECOG PS), body mass index (BMI), comorbidities, primary tumor site and characteristics, surgical procedures, postoperative treatments, and outcomes. Outcomes included overall survival (OS) duration and the incidence of perioperative complications, with a particular focus on those classified as grade 3 or higher according to the Clavien-Dindo classification [12].

Statistical analysis was conducted using EZR version 1.63 for Mac (Saitama Medical Center, Jichi Medical University, Saitama, Japan) [13]. Patients were categorized into three groups based on their preoperative GPS scores (0, 1, and 2 points). Kaplan-Meier survival analysis was performed to evaluate differences in OS across these groups. The three GPS groups were compared using the Holm method, and a log-rank trend test was conducted to assess whether the prognosis declined proportionally with increasing GPS scores.

The Cox proportional hazards model was employed to examine the association between GPS and prognosis, adjusting for potential confounding factors, including malnutrition, primary tumor site, and a number of comorbidities. Malnutrition in multivariate analysis was defined according to international malnutrition criteria, incorporating a BMI threshold of less than 20 and a C-reactive protein cutoff value of 0.3 mg/dL or higher. GPS was analyzed by comparing 0 points with 1 or 2 points, and the primary tumor site was categorized as either oropharynx/oral cavity versus hypopharynx.

Furthermore, the association between GPS and perioperative complications classified as grade 3 or higher according to the Clavien-Dindo classification was investigated. Statistical significance was set at p<0.05. Significant results are indicated with an asterisk (*) in tables, with detailed significance levels provided in the table legends.

This study adhered strictly to ethical guidelines and was approved by the hospital’s ethics committee (registration number 1201). Data collection and analysis followed all institutional and ethical standards, and patient confidentiality was maintained throughout the study.

## Results

The results are shown in Table 1. The mean age of the patients was 82.2 years, ranging from 80 to 87 years. The majority were male, 19 out of 22 patients (86%), and the ECOG PS for all patients was either 0 or 1. The mean BMI was 21.14. Comorbidities were present in 20 (90%) patients. The primary sites included hypopharyngeal cancer in 12 (54.5%) patients, oral cancer in nine (40.9%) patients, and oropharyngeal cancer in one (4.5%) patient. Clinically, stage IV was predominant, observed in 18 (81.8%) patients, with stages II and III also noted in one (4.5%) patient and three (13.6%) patients respectively, including four (18%) cases of salvage surgery. Surgical procedures included hemiglossectomy, floor-of-mouth resection, segmental mandibulectomy, marginal mandibulectomy, extensive oropharyngeal resection, and total pharyngolaryngectomy. Postoperative treatment was required in six (27.3%) patients, all receiving simple radiation therapy.

**Table 1:** Patient characteristics (n=22) Values are presented as N (%), unless otherwise indicated. Age and BMI are shown as mean (range).

Characteristics	
Mean Age	82.2 (80-87)
Gender	
Male	19 (86.4)
Female	3 (13.6)
PS 0-1	22 (100)
Mean BMI	21.14 (15.4-27.7)
Comorbidities	
Dementia	2 (9.1)
Hypertension	7 (31.8)
Diabetes	4 (18.2)
Heart Disease	4 (18.2)
Anticoagulant Use	4 (18.2)
Chronic Renal Failure	12 (54.5)
Pulmonary Disease	2 (9)
Multiple Cancers	1 (4.5)
Primary Tumor Sites	
Oral Cancer	9 (40.9)
Oropharyngeal Cancer	1 (4.5)
Hypopharyngeal Cancer	12 (54.5)
Clinical Staging	
Stage Ⅱ	1 (4.5)
Stage III	3 (14)
Stage IV	18 (81.8)
Type of Treatment	
Primary Treatment	18 (82)
Salvage Surgery	4 (18)
Surgical Procedures	
Hemiglossectomy	2 (9.1)
Floor-of-Mouth Resection	1 (4.5)
Segmental Mandibulectomy	4 (18.2)
Marginal Mandibulectomy	2 (9.1)
Extensive Oropharyngectomy	1 (4.5)
Total Laryngopharyngectomy	12 (54.5)
Bilateral Neck Dissection	14 (63.6)
Postoperative Radiation Therapy	6 (27.2)

**Figure 1:**
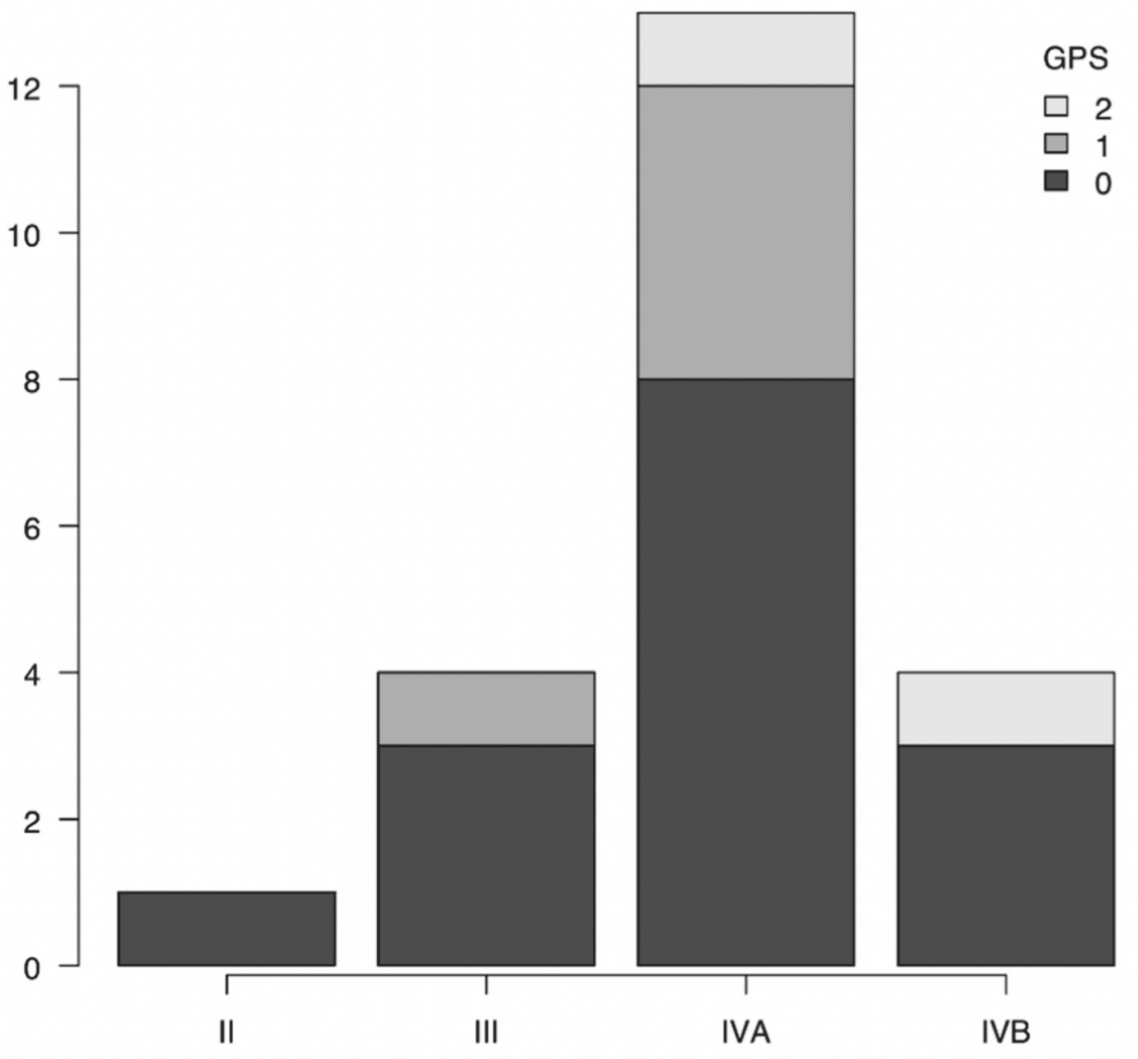
Distribution of Glasgow Prognostic Score (GPS) by cancer stage in elderly patients In this study, GPS 2 was observed only in patients with stage IV cancer; however, this observation does not imply a statistically significant association.

**Figure 2:**
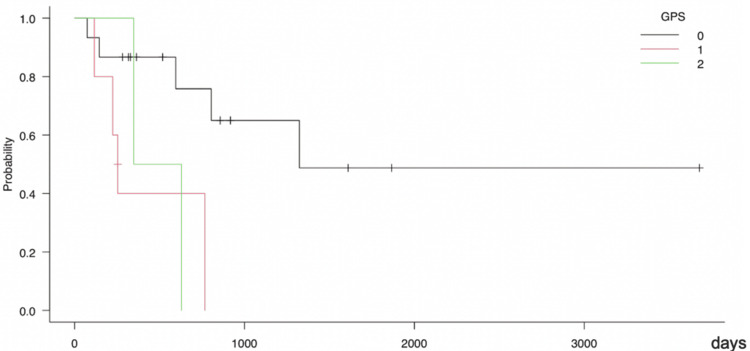
Kaplan-Meier curves by the GPS The mean observation period is 555 days. As the GPS score increases, the survival rate tends to decrease. Holm method: p-value = 0.0171; log-rank trend test: p-value = 0.016; GPS: Glasgow Prognostic Score

GPS 0 was recorded in all stages, while GPS 2 was only seen in stage IV (Figure 1). Kaplan-Meier curves by the GPS showed that the higher the GPS score, the lower the survival rate, with an average observation period of 555 days (Figure 2).

Multivariate analysis using the Cox proportional hazards model identified the GPS and primary site (the prognosis in hypopharyngeal cancer appears to be better) as independent prognostic factors (Table 2).

**Table 2:** Multivariate analysis (Cox proportional hazards model) Primary tumor site (oral/oropharynx vs. hypopharynx, *p<0.05) and GPS (0 points vs. 1 or 2 points, **p<0.05) were identified as independent prognostic factors. GPS: Glasgow Prognostic Score

	Hazards Ratio	95% CI	p-value
Malnutrition	1.005	0.1564-6.452	0.99620
Primary tumor site	0.05359	0.00461-0.6229	0.01938*
Number of comorbidities	0.54840	0.25330-1.187	0.12750
GPS	3.146	1.11500-8.876	0.03037*

**Figure 3:**
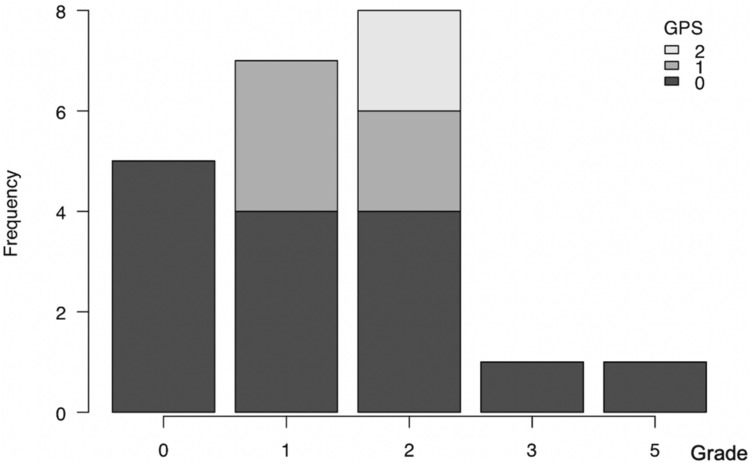
Frequency of complications by Clavien-Dindo classification

**Figure 4:**
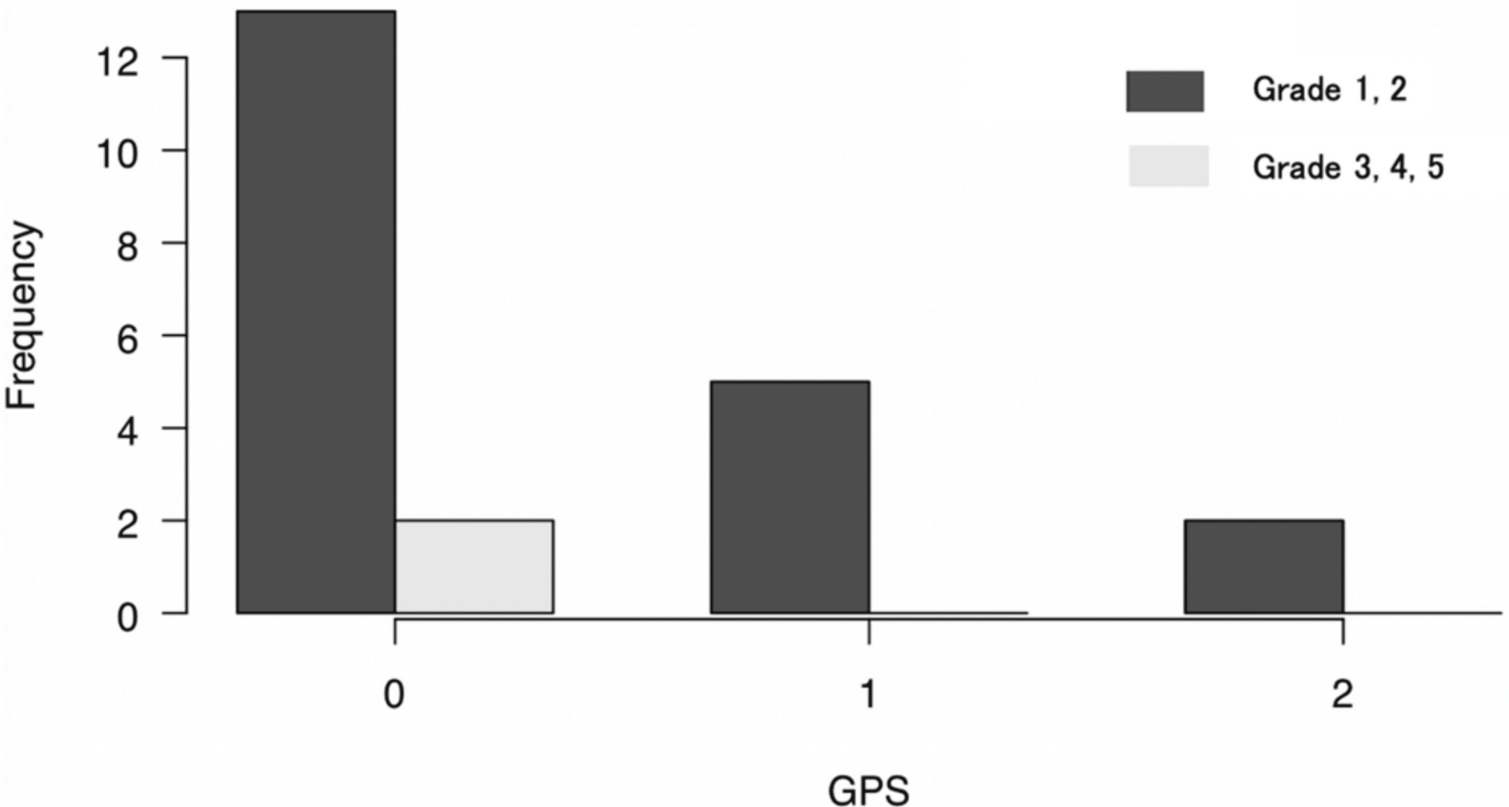
Frequency of complications by GPS No association was found between GPS and grade 3 or higher complications.

Clinically significant complications of grade 3 or higher occurred in two cases: one case of grade 3b (thrombus formation in the free flap requiring thrombectomy) and one case of grade 5 (catheter-related bloodstream infection) (Figure 3). There was no significant association between GPS and complications of grade 3 or higher (p=0.783, Figure 4).

## Discussion

The GPS is a simple indicator that evaluates inflammation and nutrition. This study aims to evaluate the effectiveness of the GPS in assessing the suitability of reconstructive surgery with free tissue transfer in elderly patients. Our results suggest that a GPS score of 0 is associated with a favorable prognosis, making it a good indication for proceeding with treatment. However, we found no correlation between the GPS, tumor stage, or postoperative complications based on the results in Figure 1 and Figure 4, highlighting the need for the development of more precise evaluation methods.

To assess the functional capacity of elderly patients with significant individual differences, National Comprehensive Cancer Network (NCCN) guidelines [14] recommend comprehensive evaluations such as CGA and G8 [6,15]. Bozec et al. conducted a retrospective cohort study on head and neck cancer patients aged 70 and above [16], finding that individualized comprehensive care based on CGA improved survival rates. Specifically, patients who received rehabilitation and nutritional management based on CGA showed better postoperative survival rates.

However, CGA implementation presents several challenges [4,5]: the need for evaluations by experts taking 1.5-2 hours (complexity and time), variation in implementation methods making standardization difficult (lack of standardization), difficulty in obtaining patient cooperation due to fatigue or cognitive decline (patient cooperation), and the requirement for multidisciplinary cooperation, which is challenging in resource-limited facilities (resource constraints).

Therefore, CGA implementation requires significant resource and time investment and can prove demanding both for the facility and the patients. Even if CGA cannot be implemented, understanding which CGA elements are essential for improving prognosis and determining suitable surgical options is crucial. Sourdet et al. [17] reported that performing CGA on 384 cancer patients with already determined treatment plans led to treatment changes in 64 (16.7%) patients. Multivariate analysis showed that assessments of physical function, cognitive function, and nutritional status were particularly associated with treatment changes. Bozec et al.'s study also indicated that effective intervention in rehabilitation and nutritional management through CGA contributed to improved prognosis [16]. Patient evaluation in these three areas and emphasizing intervention in rehabilitation and nutritional management in clinical practice is important as it seems to result in better outcomes.

As previously mentioned, nutritional management is a significant pillar of this approach. The GPS, while a convenient prognostic tool, does not always reflect nutritional status accurately. The serum albumin level included in the GPS evaluation is influenced by inflammation, dehydration or fluid ­overload, and liver and kidney function and does not always correlate with weight loss or energy deficiency [18]. Additionally, organizations such as the American Society for Parenteral and Enteral Nutrition (ASPEN) fully negate serum albumin as a nutritional indicator [19,20].

To address the limitations of the GPS relying on albumin, the Global Leadership Initiative on Malnutrition (GLIM) criteria should be used [21]. The nutritional assessment methods had not been standardized until 2018 when malnutrition diagnosis based on GLIM criteria has become the standard. GLIM, created by four societies, is the first international malnutrition standard including inflammation, and it provides a more comprehensive assessment than the GPS, which uses only albumin and CRP. GLIM criteria assess weight loss, muscle mass loss, nutritional intake, and inflammation, allowing for a comprehensive nutritional assessment. Several studies have reported that assessments based on GLIM criteria are associated with prognosis [18,19] and that they can optimize CGA [22].

A combination of the CGA and GLIM criteria would be the most effective and reliable in determining survival benefits and predicting complications in elderly patients. Unfavorable results of these assessment methods suggest that patients would benefit less or not at all from surgeries. Further studies focused on these assessments are expected.

The limitations of this study are its retrospective nature and small population. Moreover, the current GLIM criteria could not be included in the study. This study demonstrated that the GPS is a useful prognostic indicator when considering reconstructive surgery involving free tissue transfer in elderly patients. However, further research is needed to determine the best evaluation methods, including comparative studies of the CGA and GLIM criteria with the GPS. Future prospective large-scale studies should strive to solidify these findings and streamline the methods to make them more viable in the clinical setting. It needs to be clarified which CGA domains with the GLIM criteria are related to prognosis and prediction of complications.

## Conclusions

No established guidelines exist for treatment intensity in elderly patients with significant individual differences. Our retrospective analysis showed that the GPS is a useful prognostic indicator when considering reconstructive surgeries in the elderly. However, comprehensive evaluations, including CGA and nutritional assessments using the GLIM criteria, seem to have the potential to be significantly more effective and reliable.
